# Tissue culture-induced transpositional activity of *mPing *is correlated with cytosine methylation in rice

**DOI:** 10.1186/1471-2229-9-91

**Published:** 2009-07-15

**Authors:** Frédéric Ngezahayo, Chunming Xu, Hongyan Wang, Lily Jiang, Jinsong Pang, Bao Liu

**Affiliations:** 1 Key Laboratory of Molecular Epigenetics of MOE and Institute of Genetics and Cytology, Northeast Normal University, Changchun 130024, PR China; 2 Ecole Normale Supérieure, B.P. 6983 Bujumbura, Burundi

## Abstract

**Background:**

*mPing *is an endogenous MITE in the rice genome, which is quiescent under normal conditions but can be induced towards mobilization under various stresses. The cellular mechanism responsible for modulating the activity of *mPing *remains unknown. Cytosine methylation is a major epigenetic modification in most eukaryotes, and the primary function of which is to serve as a genome defense system including taming activity of transposable elements (TEs). Given that tissue-culture is capable of inducing both methylation alteration and *mPing *transposition in certain rice genotypes, it provides a tractable system to investigate the possible relationship between the two phenomena.

**Results:**

*mPing *transposition and cytosine methylation alteration were measured in callus and regenerated plants in three rice (ssp. *indica*) genotypes, V14, V27 and R09. All three genotypes showed transposition of *mPing*, though at various frequencies. Cytosine methylation alteration occurred both at the *mPing*-flanks and at random loci sampled globally in callus and regenerated plants of all three genotypes. However, a sharp difference in the changing patterns was noted between the *mPing*-flanks and random genomic loci, with a particular type of methylation modification, i.e., CNG hypermethylation, occurred predominantly at the *mPing*-flanks. Pearson's test on pairwise correlations indicated that *mPing *activity is positively correlated with specific patterns of methylation alteration at random genomic loci, while the element's immobility is positively correlated with methylation levels of the *mPing'*s 5'-flanks. Bisulfite sequencing of two *mPing*-containing loci showed that whereas for the immobile locus loss of CG methylation in the 5'-flank was accompanied by an increase in CHG methylation, together with an overall increase in methylation of all three types (CG, CHG and CHH) in the *mPing*-body region, for the active locus erasure of CG methylation in the 5'-flank was not followed by such a change.

**Conclusion:**

Our results documented that tissue culture-induced *mPing *activity in rice ssp. *indica *is correlated with alteration in cytosine methylation patterns at both random genomic loci and the elements' flanks, while the stability of *mPing *positively correlates with enhanced methylation levels of both the flanks and probably the elements *per se*. Thus, our results implicate a possible role of cytosine methylation in maintaining *mPing *stability under normal conditions, and in releasing the element's activity as a consequence of epigenetic perturbation in a locus-specific manner under certain stress conditions.

## Background

Transposable elements (TEs) are sequences capable of changing their physical locations in their host genomes [1,2]. TEs are ubiquitous constituents of all eukaryotic genomes so far investigated, and particularly abundant in plants, where they can occupy more than 80% of the genomic sequences [3,4]. TEs are composed of RNA retrotransposons (class I) and DNA transposons (class II). Whereas RNA retrotransposons require a reverse-transcription step to transpose in a "copy-and-paste" manner, DNA transposons transpose via a "cut-and-paste" mode [3]. Therefore, whereas retrotransposons usually reach very high copy numbers, DNA transposons often retain low copies [5]. One exception to this general rule is the miniature inverted-repeat TEs (MITEs), which are DNA transposons, yet they can reach high copy numbers in the range of thousands [3].

MITEs have been classified into two superfamilies, *Tourist*-like and *Stowaway*-like, based on the similarity of their terminal inverted repeats (TIRs) and target site duplications (TSDs) [3]. The possible roles of MITEs in the evolution of structure and function of plant genes were implicated by their preferential association with low-copy, genic regions [6,7], and shown by several documented cases wherein the presence *vs*. absence of a particular MITE being correlated with expression states of the genes in question [4-9].

Whole genome data mining in rice (*Oryza sativa *L.) revealed that MITEs are major components of interspersed repetitive sequences of the genome [10,11]. Nonetheless, to date only one MITE family, called *mPing*, has been experimentally demonstrated as transpositionally active in the rice genome [12-14], though some other types of DNA transposons, e.g., *nDart *[15] was also shown as active. *mPing *is a 430 bp DNA sequence with terminal inverted repeats or TIRs (15 bp) and target site duplications or TSDs (TAA or TTA) typical of a *Tourist*-like MITE [12-14]. Albeit being exceptionally low in copy number compared with other characterized MITE families in plants [3,16], *mPing *can be effectively mobilized by several stressful conditions like tissue culture [12,14], irradiation [13], hydrostatic pressurization [17], and interspecific hybridization [18]. Because *mPing *has no coding capacity, the transposase (TPase) required to catalyze its transposition is provided *in trans *by related autonomous element(s) [3,12,16]. Based on sequence homology, co-mobilization and transpositional capacity in a non-host genome (*Arabidopsis thaliana*) with *mPing*, both of the *mPing*-related, transposase-encoding elements, *Ping *and *Pong*, are demonstrated as TPase donors for *mPing*, though *Pong *appeared to have a higher mobilizing capacity [12,14,19].

Cytosine DNA methylation is an important epigenetic marker that exists in most animal and plant genomes. Whereas in mammalian animals this modification occurs almost exclusively at the CG dinucleotides, cytosines of any sequence context including CG, CHG and CHH (H is any base other than G) can be methylated in plants [20,21]. Cytosine methylation has been proposed to have diverse cellular functions in eukaryotes, but its primary role was believed to serve as a genome surveillance and defense system such as taming of TEs [22,23]. Indeed, close correlations between TE activity and its methylation states were documented in several plants including maize [24-27], rice [28-30], and particularly *Arabidopsis *[31,32]. More recent studies in *Arabidopsis *have further strengthened the relationship and even enabled the establishment of causal links between TE activity and its DNA methylation states. For example, it was found in *Arabidopsis *that silencing of an introduced retrotransposon (*Tto1*) was caused by hypermethylation of the element, and genome-wide hypomethylation (in the *ddm1 *mutant background) results in its reactivation and transposition [33]. The *ddm1 *mutation in *Arabidopsis*, which results in genome-wide methylation reduction by 70% [34], has caused transposition of an otherwise dormant endogenous CACTA transposon, and produced a spectrum of new insertions [31]. Furthermore, it was demonstrated that multiple TEs were activated in single, double and triple loss-of-function mutants of the various DNA methyltransferases, *MET1, CMT3 *and *DRM2 *in *Arabidopsis*,which have provided unequivocal evidence for the deterministic role of DNA methylation in controlling both transcriptional and transpositional activities of specific families of TEs [35-37]. These studies also revealed that methylation of CG and CHG play both overlapping and distinct functional roles in maintaining transcriptional quiescence and transpositional immobility of specific types of TEs [35].

Although stress-induced mobility of *mPing *has been studied extensively both in its native host (rice) [14,17,18] and in an alien genome (*Arabidopsis*) [19], it is unclear whether cytosine methylation plays any role in the element's activity. As a first step to explore possible epigenetic mechanisms underlying the regulation of *mPing *activity, we tested whether alteration of status of cytosine methylation of random genomic loci and regions immediately flanking the element copies might be associated with the element's transposition in rice. To address this issue, we employed tissue culture of three rice ssp. *indica *cultivars in which *mPing *can be efficiently mobilized and marked alteration in cytosine methylation of various types occurs. We report that statistically meaningful correlations exist between *mPing *activity and alteration in cytosine methylation at random genomic loci, and between *mPing *stability and heavy methylation status of *mPing per se *as well as regions immediately flanking the element. We propose that cytosine methylation likely plays an important role in maintaining *mPing *stability under normal conditions, and in releasing the element's activity as a consequence of perturbation in the epigenetic modification by certain stress conditions like tissue culture.

## Results

### Tissue culture-induced *mPing *transposition

**Figure 1:**
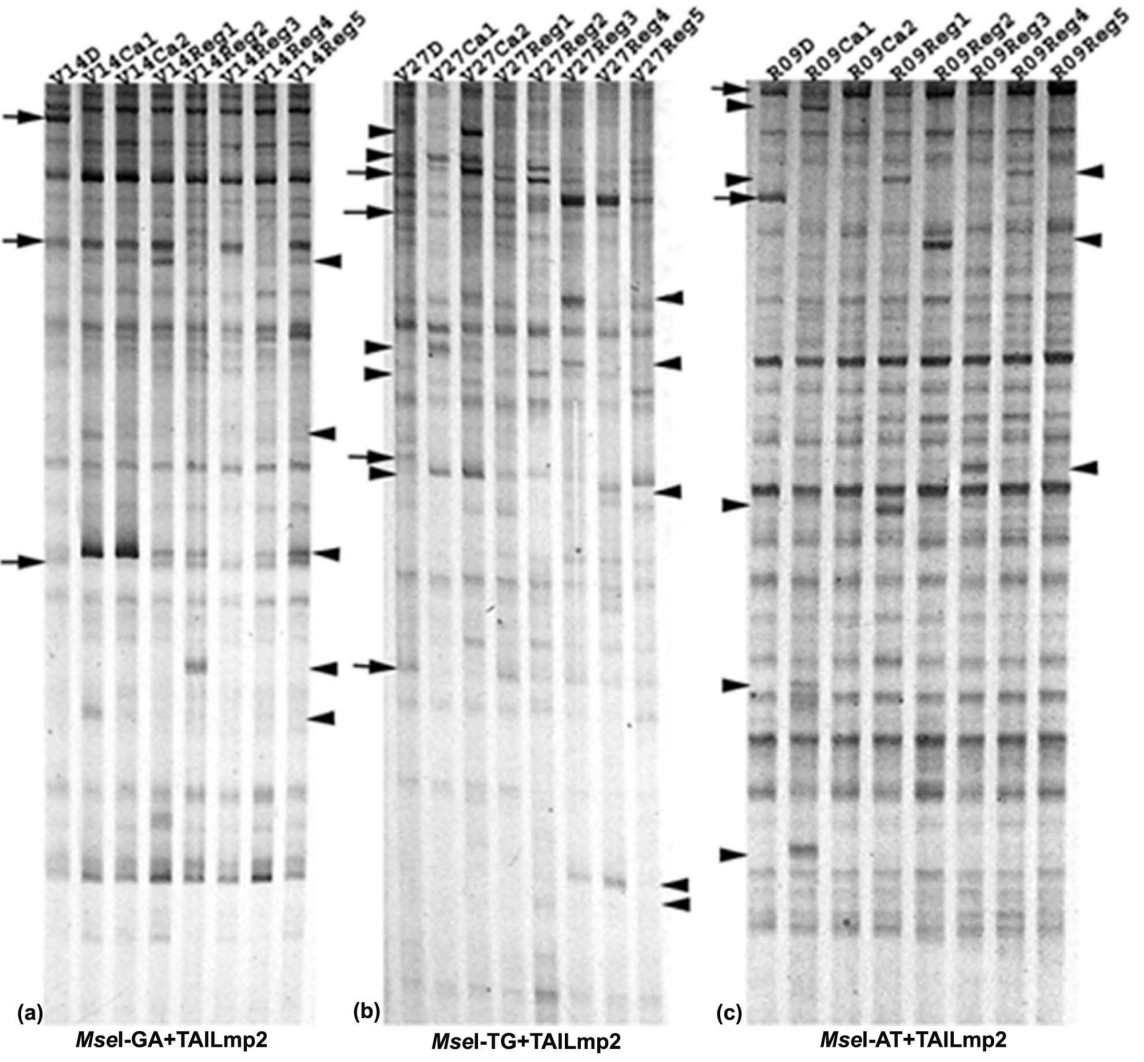
**Examples of transposon display (TD) profiles showing the tissue culture-induced *mPing *activity in the three rice ssp. *indica *cultivars**. (a), (b) and (c) are profiles of cultivars V14, V27 and R09, respectively. The labeling V14D, V27D and R09D are the donor seed-plants of the three genotypes; V14Ca1–2, V27Ca1–2 and R09Ca1–2 are pooled calli; and, V14Reg1–5, V27Reg1–5 and R09Reg1–5 are regenerated plants. Arrowheads and arrows refer, respectively, to excisions and insertions of *mPing*. Primer combinations are indicated at bottom of the profiles.

Transposon display (TD) analysis was performed using combinations of *Mse*I-adaptor-primers with two consecutive *mPing*-specific primers at the 5' end (named as TAILmp-1 and -2) to assess the transpositional activity of *mPing *in calli and regenerants of the three rice ssp. *indica *genotypes. Because amplification by using each of the *Mse*I adaptor-primers alone produced no resolvable bands in the gel-running range (200–1000 bp), all resolvable bands on the TD profiles should have resulted from hetero-amplifications, i.e., a *Mse*I-adaptor primer plus the *mPing*-specific primer (see Additional file 1). As exemplified in Figure 1, for a given genotype, three types of bands were resolvable: (i) monomorphic bands uniformly present in the donor plant and its corresponding calli and regenerants, (ii) polymorphic bands present in the donor plant but disappeared in calli and/or regenerant(s), and (iii) polymorphic bands that were novel in calli and/or regenerant(s). These three types of bands should correspond, respectively, to static, excised and newly inserted *mPing *copies in the calli and/or regenerant(s) relative to their donor seed-plants in a given genotype (Figure 1). Indeed, by isolating representatives of these three types of bands as templates, and using the same *Mse*I-adaptor primer together with the third *mPing*-specific primer (named TAILmp3) that is further internal to the two primers (TAILmp1 and 2), mentioned above, authenticity was validated in each case as judged by the expected band size differences in agarose gels (data not shown).

**Figure 2:**
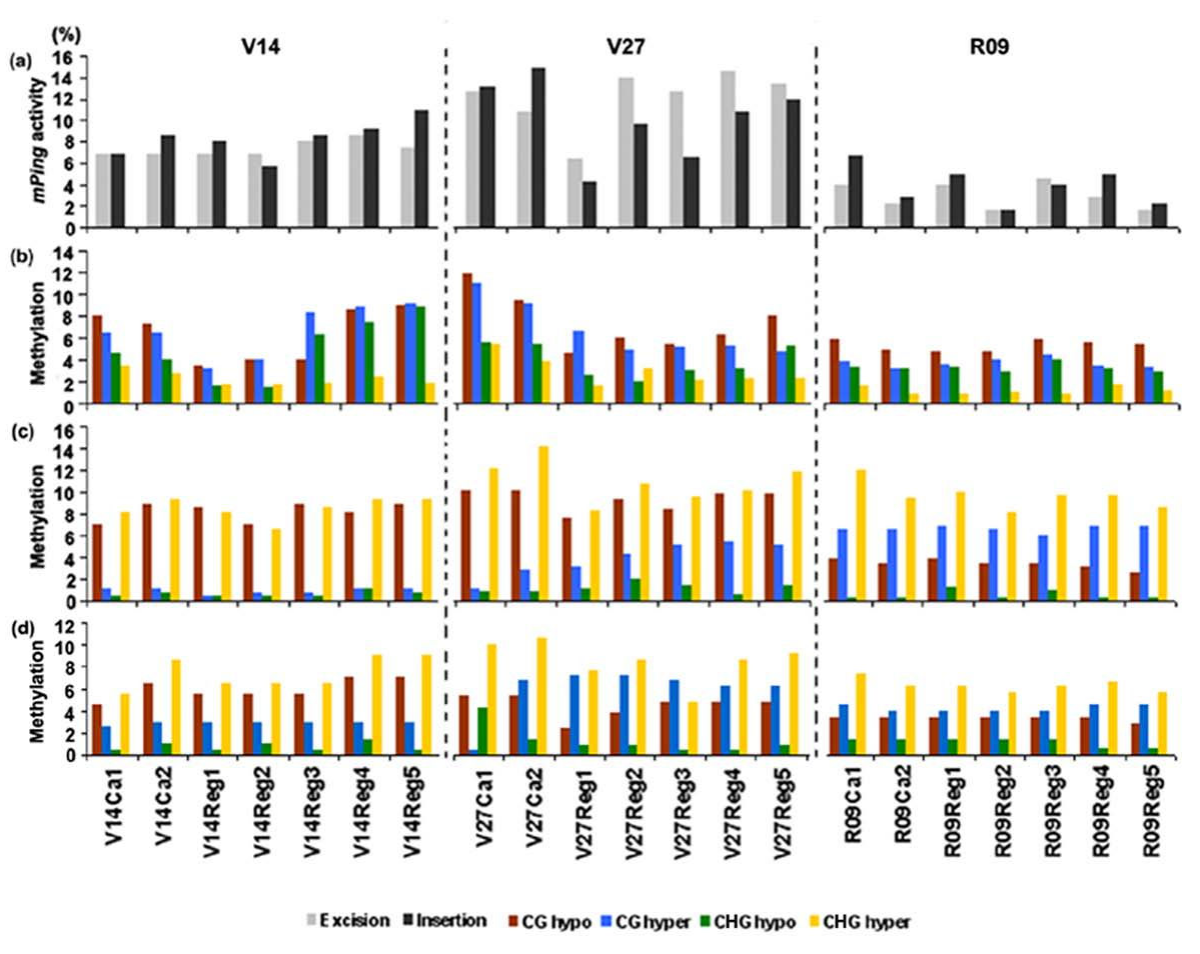
**Summary of the tissue culture-induced *mPing *activity and alteration in cytosine methylation in the three rice ssp. *indica *cultivars**. (a) *mPing *activity as being reflected by the frequencies of excision and insertion in each genotype; (b) the four types of alteration in cytosine methylation at the CCGG sites of random genomic loci assessed by MSAP; (c) The four types of alteration in cytosine methylation at the CCGG sites of the 5' immobile *mPing*-flanking regions assessed by TMD; (d) The four types of alteration in cytosine methylation at the CCGG sites of the 3' immobile *mPing*-flanking regions assessed by TMD. *mPing *excisions and insertions, as well as the four types of methylation alteration (CG hypo-, CG hyper-, CHG hypo- and CHG hypermethylation) are indicated at bottom of the figure.

Although calli and regenerants of all three genotypes showed high mobility of *mPing*, both excisions and insertions varied markedly among them (Figure 1), with V27 and V14 showed markedly higher numbers than those of R09 (Figure 2a). More than 30 TD bands, each showed at least one missing event in calli and/or regenerants relative to their donor plant for a given genotype (Figure 1), were isolated and sequenced, but only 10 distinct loci (the rest being redundant) were found to contain at their 5' terminus portions of the *mPing *sequence, as expected for *mPing*-containing loci. In addition, by taking advantage of the draft genome sequence of the *indica *rice cultivar 93–11 [38], locus-specific primers were designed for each of the loci, and the corresponding putative "*mPin*g-empty loci" were also amplified from the donor seed-plant, and sequenced (Additional file 2). Pairwise sequence comparisons confirmed that they represent bona fide *mPing *excisions, though none of the excisions had left behind any footprints (see Additional file 2). By the same rational, 30 different TD bands that were novel in calli and/or regenerant(s) relative to their donor plant for a given genotype (Figure 1) were also isolated and sequenced. Sequence analysis indicated that these 30 novel TD bands all contained at their 5' terminus the expected portion of the *mPing *sequence with typical 15 bp terminal inverted repeats (TIRs) and target site duplications (TSD) of TAA or TTA, suggesting they were *de novo mPing *insertions induced by tissue culture. Again, locus-specific primers flanking each of the "*mPing *insertion-loci were designed based on the 93–11 draft genome sequence, and used to amplify the "complete" loci (i.e., *mPing *with both flanks). Further sequencing of the complete loci confirmed that they all were bona fide *mPing de novo *insertions in the calli and/or regenerants (see Additional file 3). A Blast N analysis of these insertion loci with the annotated genome draft sequence of 93–11 indicated that all insertions mapped to unique- or low-copy regions (see Additional file 3). This is consistent with targeting propensity of *mPing *insertions induced by other stress conditions [39].

### Tissue culture-induced alteration in cytosine methylation at random loci across the genome revealed by methylation-sensitive amplified polymorphism (MSAP) analysis

*Hpa*II and *Msp*I are a pair of isoschizomers that recognize the same restriction site (5'-CCGG) but have differential sensitivity to certain methylation states of the two cytosines: *Hpa*II will not cut if either of the cytosines is fully (double-strand) methylated, whereas *Msp*I will not cut if the external cytosine is fully- or hemi- (single-strand) methylated [40]. Thus, for a given DNA sample, the full methylation of the internal cytosine, or hemi-methylation of the external cytosine, at the assayed CCGG sites can be unequivocally identified by MSAP [41-45]. For clarity, we hereby refer to these two types of patterns as CG and CHG methylations, respectively.

**Figure 3:**
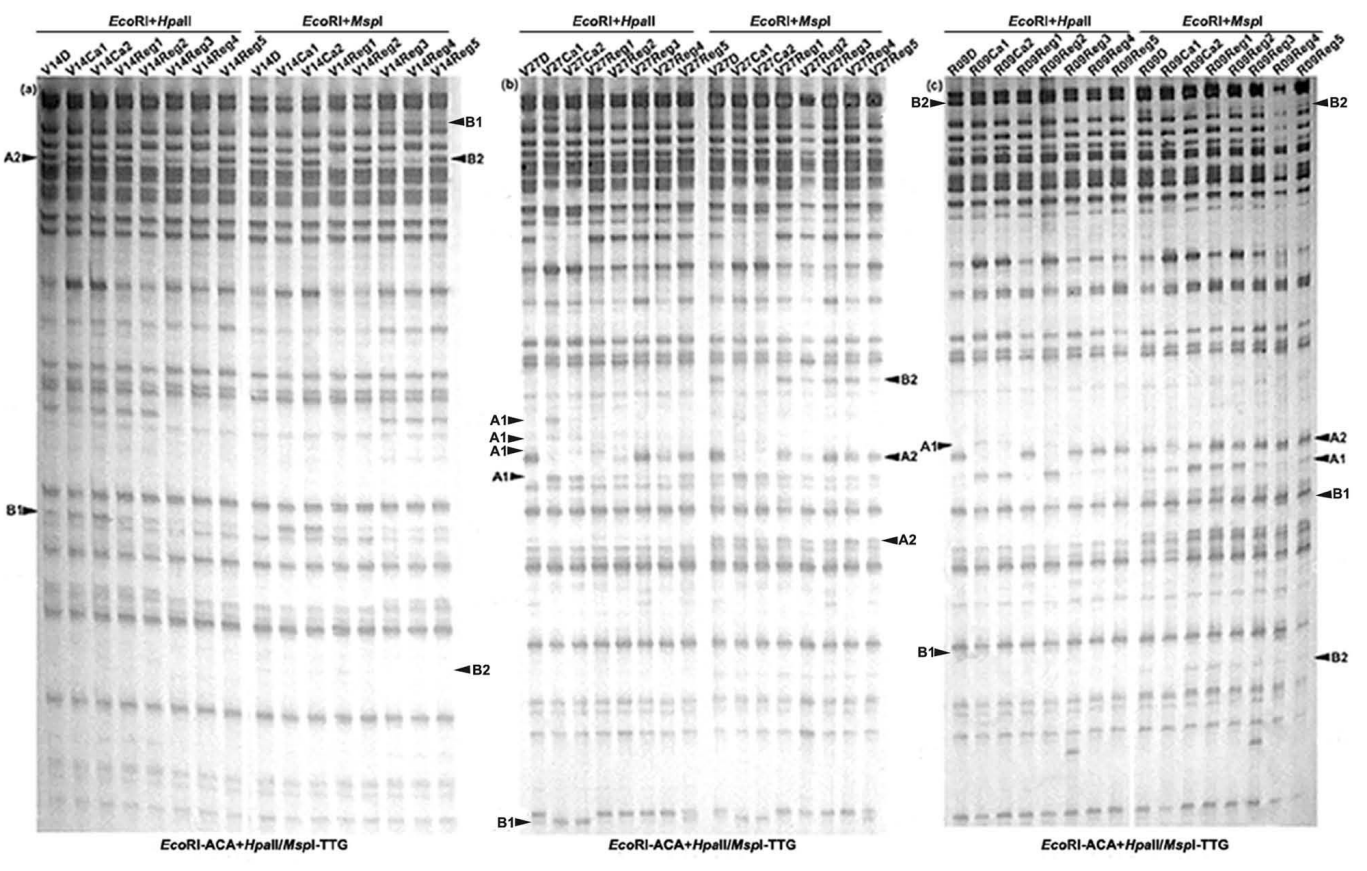
**Examples of MSAP profiles showing the tissue culture-induced alteration in cytosine methylation at the CCGG sites of random genomic loci in the three rice ssp. *indica *cultivars**. (a), (b) and (c) are profiles of cultivars V14, V27 and R09, respectively. The labeling V14D, V27D and R09D are the donor seed-plants of the three genotypes; V14Ca1–2, V27Ca1–2 and R09Ca1–2 are calli; and, V14Reg1–5, V27Reg1–5 and R09Reg1–5 are regenerated plants. The four types of alteration in cytosine methylation pattern at the CCGG sites are indicated as A1 – CG hypomethylation, A2 – CG hypermethylation, B1 – CHG hypomethylation, and B2 – CHG hypermethylation. The primer combinations are indicated at bottom of the profiles.

By using 17 pairs of *Eco*RI + *Hpa*II/*Msp*I primer combinations (see Additional file 1), 696, 731 and 706 clear and reproducible MSAP bands (between two technical replicates) were scored for each of the genotypes, V14, V27 and R09, respectively. Relative to the donor plant, the MSAP profiles of calli and regenerants revealed the occurrence of four types of cytosine methylation alteration at the CCGG sites (see Additional file 4), as exemplified in Figure 3. These are: CG hypomethylation (marked as A1), CG hypermethylation (marked as A2), CHG hypomethylation (marked as B1), and CHG hypermethylation (marked as B2). Although some difference in terms of alteration frequencies existed among the three genotypes, the general trend of alteration of all four types is remarkably similar across genotypes (Figure 2b), which led to the following two generalizations: (1) between the two types of cytosines, CG and CHG, more alteration occurred at the CG sites than the CHG sites; (2) among all four types of alteration patterns, the mostly occurred type is CG hypomethylation, followed by CG hypermethylation and then CHG hypomethylation, with CHG hypermethylation being the least occurred type (Figure 2b). To obtain some information regarding the genomic location and possible functional relevance of the sequences underlying the methylation alteration, a subset of 29 MSAP bands representing the various types were isolated and sequenced (see Additional file 5). A Blast N analysis showed that these fragments mapped to 11 of the 12 rice chromosomes (except chromosome 8). A Blast X analysis indicated that 16 bands (E2, E6, E7, E9, E13, E21, E22, E29, E34, E46, E49, E54, E57, E73, E76 and E77) bear meaningful homology to hypothetical proteins with diverse functions, one (E35) to an unknown protein, one (E24) to a *En/Spm *subclass transposon protein, and one (E58) to a *Ty1-copia *retrotransposon, while the rest 10 showed no significant similarity to the available database sequences (see Additional file 5). The sequence analysis also showed that 10 bands contained internal (and hence methylated) CCGG sites (see Additional file 5).

### Tissue culture-induced alteration in cytosine methylation at *mPing*-flanking regions revealed by transposon-methylation display (TMD)

**Figure 4:**
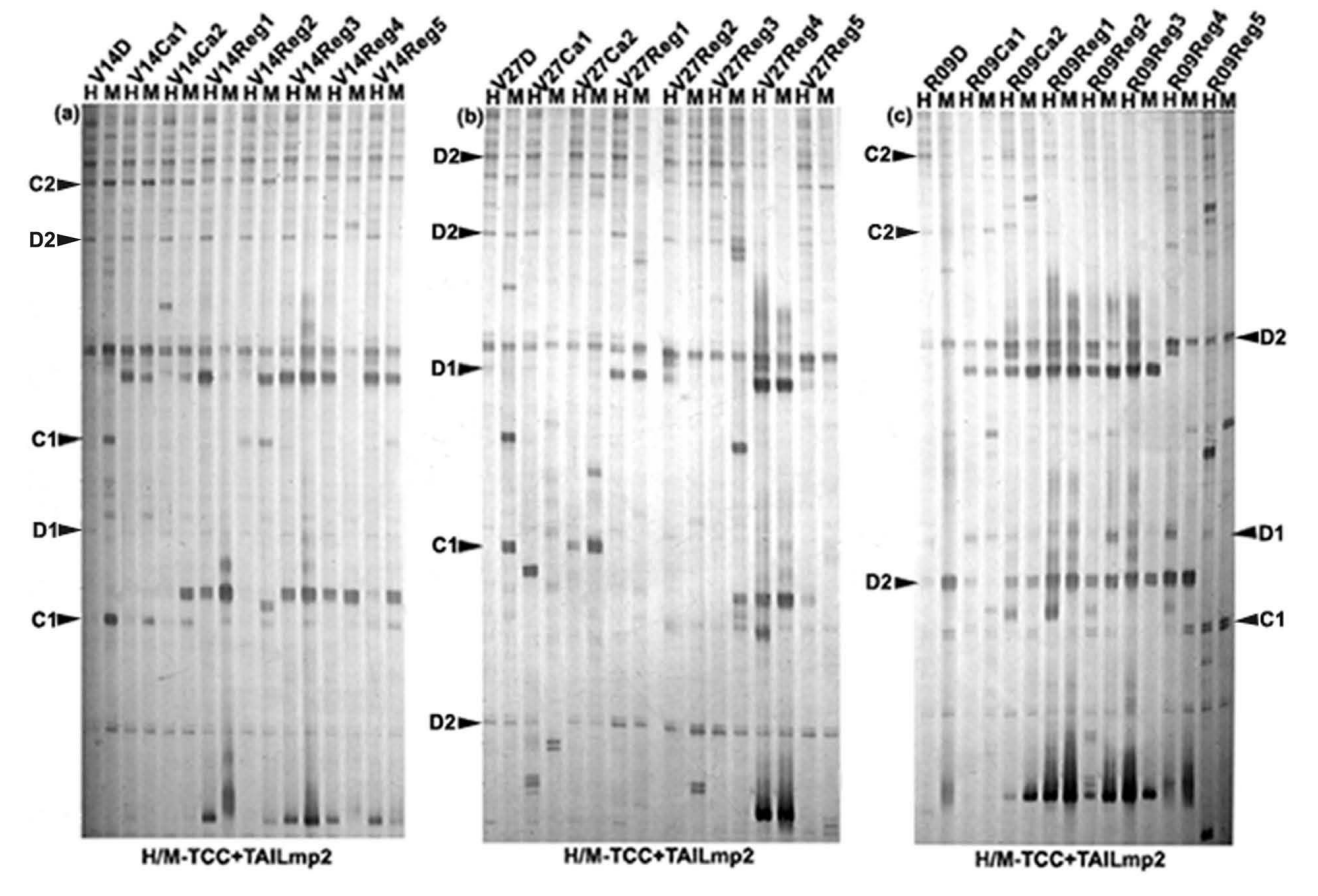
**Examples of transposon (*mPing*)-methylation display (TMD) profiles showing the tissue culture-induced alteration in cytosine methylation at the CCGG sites of the 5' *mPing*-flanking regions of the three rice ssp. *indica *cultivars**. (a), (b) and (c) are profiles of cultivars V14, V27 and R09, respectively. Similar TMD profiles were obtained for the 3' *mPing*-flanking regions. The labeling of the four types of alteration in cytosine methylation at the immobile *mPing*-flanking regions are indicated as C1 – CG hypomethylation, C2 – CG hypermethylation, D1 – CHG hypomethylation, and D – CHG hypermethylation. The primer combinations are indicated at bottom of the profiles.

To assess methylation levels in the genomic regions immediately flanking the *mPing *copies, we performed transposon (*mPing*)-methylation display (TMD) assay. TMD is a modified version of transposon-display (TD) by substituting the original *MseI *digestion with methylation-sensitive *Hpa*II/*Msp*I-digestions (see Methods). Another modification we made here was that *mPing*-specific primers targeting at both the 5' and 3' ends were included (Methods). To rule out confounding polymorphic bands due to *mPing *transpositions (excisions or insertions), only those changing TMD patterns that appeared in one but not both of the digestions (*Hpa*II and *Msp*I) were scored for a given genotype (see Additional file 4). Therefore, it should be pointed out that only the genomic regions flanking the immobile *mPing *copies were amenable to the assay. As in MSAP, the changing methylation patterns revealed by TMD were also divided into four major types, CG hypomethylation (C1), CG hypermethylation (C2), CHG hypomethylation (D1) and CHG hypermethylation (D2) (see Additional file 4), as exemplified in Figure 4. We found that in general the 5' and 3' immobile *mPing *– flanking regions showed similar trend of alteration in all four types of methylation patterns, though differences are evident for a given type of alteration within a genotype (Figure 2c, d). If comparing the methylation pattern alteration of the immobile *mPing*-flanking regions (Figure 2c, d) with those of random genomic loci (revealed by MSAP, Figure 2b), a striking feature of the immobile *mPing*-flanking regions is that they showed markedly higher frequencies of CHG hypermethylation in all three genotypes (Figure 2c, d).

### Correlation between *mPing *activity and alteration in cytosine methylation at random genomic loci

To test if there exists any intrinsic correlation between tissue culture-induced *mPing *activity and alteration in cytosine methylation patterns at the CCGG sites of random genomic loci across the genome, various correlation coefficients between these two "characters" were calculated. We found that when excisions and insertions were considered together as "*mPing *activity", no correlation between alteration in cytosine methylation at random genomic loci (based on the MSAP data) and *mPing *activity was found irrespective of whether the three genotypes were considered separately or together (data not shown). However, when excisions and insertions were considered separately on a per-genotype basis, and methylation alteration being dissected into specific types, i.e., CG or CHG, the correlation coefficients were statistically significant in four cases. These are: (1) between *mPing *insertions and CHG hypomethylation in genotype V14 (r = 0.806, P < 0.05); (2) between *mPing *insertions and CG hypomethylation in genotype V27 (r = 0.843, P < 0.05); (3) between *mPing *insertions and CHG hypomethylation in genotype V27 (r = 0.767, P < 0.05), and; (4) between *mPing *excisions and CHG hypomethylation in genotype R09 (r = 0.866, P < 0.05) (Table 1). Obviously, for a given genotype, at least one type of cytosine methylation alteration is significantly correlated with at least one aspect of *mPing *transpositional activity (excision or insertion). If all three genotypes were considered together, the separation of excisions and insertions produced even more meaningful correlations. These are (1) between *mPing *excisions and three of the four types of methylation alteration (r values ranged from 0.488 to 0.664, P < 0.05 or 0.01), with CHG hypomethylation being the only exception, and; (2) between *mPing *insertions and each of the four types of methylation alteration (r values ranged from 0.558 to 0.728, P < 0.01) (Table 1). From this analysis, it is clear that there indeed exist statistically meaningful positive correlations between tissue culture-induced *mPing *activity and alteration in specific types of cytosine methylation patterns at random loci across the genome, but the correlations are "visible" only when (1) *mPing *activity was separated into excisions and insertions, and; (2) the methylation alteration were dissected into specific patterns.

**Table 1:** Pearson's correlation coefficient values between the four types of methylation alteration at the CCGG sites detected by MSAP and *mPing *activity in each or all three rice (ssp. *indica*) genotypes

Genotype	*mPing *activity	Different types of alteration in cytosine methylation and correlation coefficient values			
		CG Hypo-methylation	CG Hyper-methylation	CHG Hypo-methylation	CHG Hyper-methylation

V14	Excision	0.230	0.731	0.729	-0.196
	Insertion	0.543	0.723	0.806*	-0.176
V27	Excision	0.242	-0.255	0.064	0.208
	Insertion	0.843*	0.499	0.767*	0.703
R09	Excision	0.472	0.555	0.866*	-0.012
	Insertion	0.503	0.132	0.403	0.498
All	Excision	0.488*	0.510*	0.253	0.664**
	Insertion	0.728**	0.704**	0.558**	0.756**

*Significant at the 0.05 statistic level **Significant at the 0.01 statistic level

### Correlation between *mPing *immobility and cytosine methylation level at the mPing-flanking regions

If meaningful correlations exist between tissue culture-induced *mPing *activity and alteration in cytosine methylation patterns at random genomic loci from a global perspective (based on the MSAP data), then an intuitive question to ask is whether the *mPing *activity should be equally or even more correlated with cytosine methylation of the genomic regions immediately flanking the element copies. To investigate this possibility, we calculated correlation coefficients between levels of the two major types of methylation, CG and CHG, of each of the 5'- and 3'-*mPing *flanking regions detected by *mPing*-TMD and *mPing *immobility. It should be pointed out that, in contrast to the situation of random loci sampled genome-widely (described above), with TMD only *mPing *immobility (or stability) can be considered because the genomic regions flanking active *mPing *copies can not be amplified from the calli and/or regenerants (due to excision) by the TMD assay (see Methods). Nonetheless, we reasoned that if methylation status of the flanking regions plays a role in the *mPing *activity, then we would expect to find a meaningful correlation between high levels of methylation and *mPing *stability, i.e., a positive correlation should exist. Indeed, the correlation analysis (Table 2) established the following positive relationships: (1) in genotype V14, *mPing *stability correlates with two of the four types of methylation levels, i.e., CG of the 5'-flank and CHG methylation of the 5'-flank (r = 0.727 and 0.81, respectively, P < 0.05); (2) in genotype V27, *mPing *stability correlates with three of the four types of methylation levels, i.e., CG of the 5'-flank, CG of the 3'-flank and CHG of the 5'-flank (r = 0.872, 0.803 and 0.782, respectively, p < 0,05 or 0.01); (3) in genotype R09, *mPing *stability correlates with two of the four types of methylation levels, i.e., CG of the 5'-flank and CG of the 3'-flank (r = 0.856 and 0.837, respectively, p < 0,05 or 0.01); (4) when all three genotypes being considered together, *mPing *stability correlates with three of the four types of methylation levels, i.e., CG of the 5'-flank, CG of the 3'-flank and CHG of the 5'-flank (r = 0.852, 0.665 and 0.724, respectively, p < 0,05 or 0.01). A conclusion emerged from the correlation data is that whereas CG methylation of both the 5'- and 3'-flanking regions likely plays important roles in maintaining *mPing *stability, CHG methylation of only the 5'-flanking regions appeared important for the purpose (Table 2).

**Table 2:** Pearson's correlation coefficient values between *mPing *stability and cytosine methylation levels at the CCGG sites of genomic regions immediately flanking the immobile copies of *mPing *in each or all three rice (ssp. *indica*) genotypes

		Methylation level at the CCGG sites flanking immobile *mPing *copies			
		
Genotype	*mPing *stability	CG (%)		CHG (%)	
		5'-flank	3'-flank	5'-flank	3'-flank
V14	Stability	0.727*	0.355	0.861*	0.120
V27	Stability	0.872*	0.803*	0.782*	0.415
R09	Stability	0.856*	0.837*	0.474	0.586
All	Stability	0.852**	0.665**	0.724**	0.323

*Significant at the 0.05 statistic level **Significant at the 0.01 statistic level

### Cytosine methylation status of an inactive (immobile) and an active *mPing*-containing loci determined by bisulfite genomic sequencing

**Figure 5:**
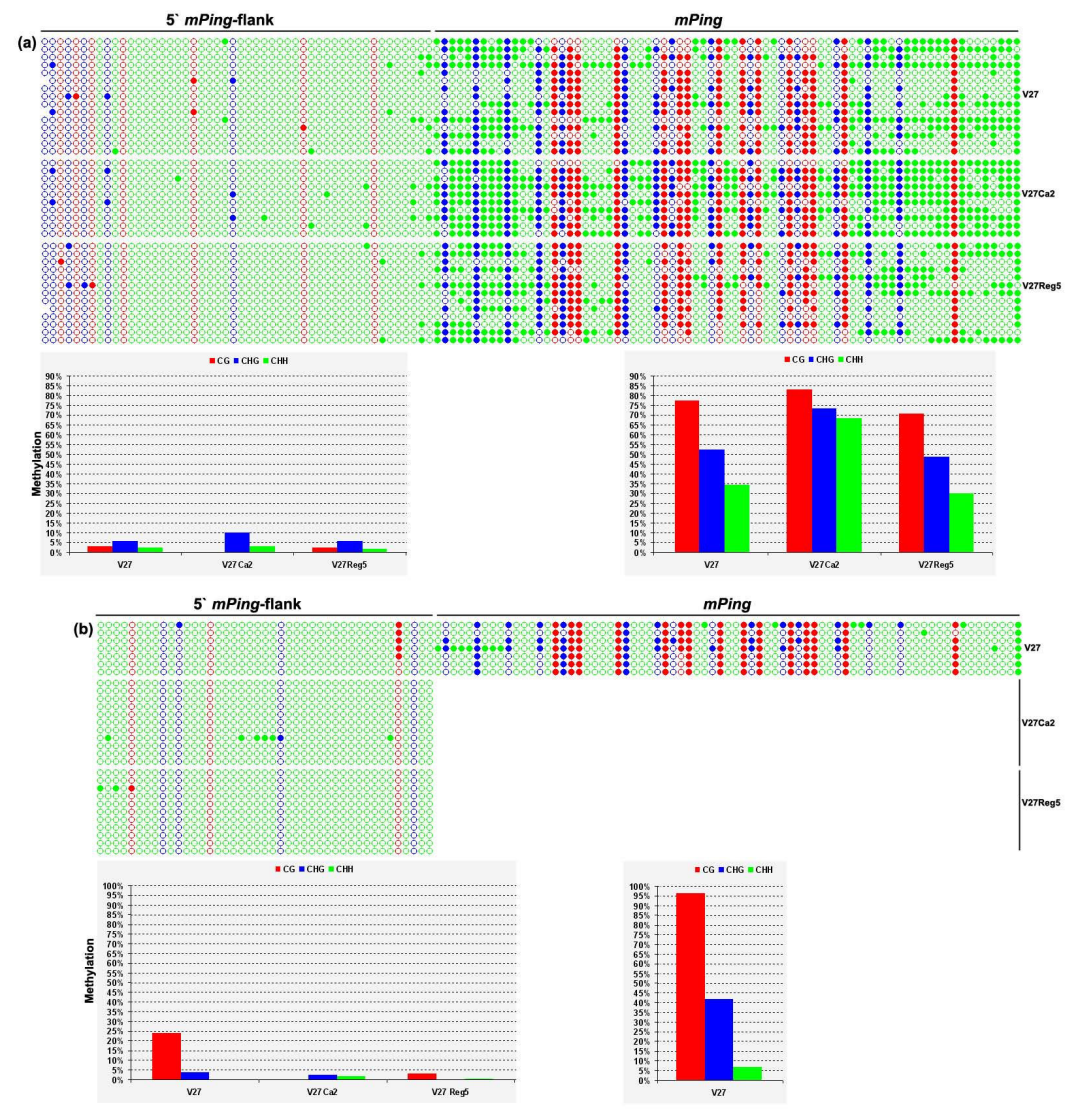
**Cytosine methylation maps and collective methylation values (in percentage) for an inactive (immobile) *mPing*-containing locus (ITDTG8) (a) and an active (excised) *mPing*-containing locus (ITDTA6) (b) in seed-plant (V27), a pool of calli (V27Ca2) and a regenerated plant (V27Reg5) of cv. V27, determined by genomic bilsulfite sequencing**. All three types of cytosines, CG (red circles), CHG (blue circles) and CHH (green circles), at the immediate 5'-flanks and portion of the *mPing *body-regions were shown in the map. Filled and empty circles denote methylated and unmethylated cytosines, respectively. The red, blue and green columns in the histograms refer to the collective methylation levels (in percentage) respectively of CG, CHG and CHH, at each part (5'-flank or *mPing*-body) of the two loci for each analyzed plant sample.

To further investigate the difference in cytosine methylation between inactive (immobile) *mPing *copies and active ones (showing excision), we determined the cytosine methylation status of portion of the *mPing *body-regions and their immediate 5' flanks by bisulfite sequencing for one locus of each kinds, ITDTG8 (inactive) and ITDTA6 (active), which were arbitrarily chosen from the TD profiles (Figure 1). We found that (1) for the inactive *mPing*-containing locus (ITDTG8), the 5'-flank was slightly methylated (< 5%) in the seed-plant for all three types of methylation, CG, CHG and CHH; the residual CG methylation (3%) was completely lost in the callus, and which was accompanied by a increase in CNG methylation (from 6% to 10%), while the residual CHH methylaiton (2%) remained unchanged; the methylation status of all three types were restored to those of the seed-plant in the regenerated plant (Figure 5a). In contrast to the situation of the 5'-flank, the *mPing *body-region at this locus was heavily methylated in CG (77%) and moderately methylated in CHG (52%) and CHH (35%) in seed-plant, and the degree of all three types of methylation in the *mPing *body-region was further increased in callus, particularly in CHG and CHH, followed by a decrease to roughly the original levels of seed-plant in the regenerated plant (Figure 5a). (2) For the active *mPing*-containing locus (ITDTA6), in the seed-plant the 5'-flank was partly methylated in CG (24%), residually methylated in CHG (4%) and non-methylated in CHH (Figure 5b); notably, this CG methylation was completely erased in the callus, and unlike the case in the immobile *mPing*-containing locus (Figure 5a), this CG hypomethylation was not accompanied by CHG hypermethylation (though very slight CHH remethylation) (Figure 5b). The *mPing *body-region at this locus in seed-plant (prior to excision) was also heavily methylated in CG (96%) which was even higher than that of the inactive copy (77%), but the methylation levels of CHG (41%) and CHH (6%) of this active *mPing *copy were markedly lower than those of the inactive copy (41% *vs*. 52% and 6% *vs*. 35%, respectively for CHG and CHH). It is not possible to analyze possible methylation changes at the *mPing *body-region of this locus during the callus stage, as it was excised. Collectively, the bisulfite genomic sequencing data suggest that methylation status of both the 5'-flanks and the body-regions of *mPing *may be important for its activity or inactivity, depending on the loci. Thus, under the tissue culture stress conditions, whereas CG hypomethylation in the element's 5'-flanks might have played a part in facilitating the excision of active *mPing *copies (Figure 5b), further enhancement in methylation at both the flanks and the element body-regions (particularly CHG and CHH; Figure 5a) might have played a critical role in fortifying stability of the immobile copies. This is consistent with the global correlation analysis between *mPing *activity and methylation alteration at random genomic loci (detected by MSAP, Table 1), and between *mPing *immobility and methylation level of the flanks (revealed by TMD, Table 2), described above.

## Discussion

It has been demonstrated that among all kinds of TEs, MITEs are most closely associated with plant genes [6-9]. This, together with their propensity to accumulate to high-copy numbers (relative to other types of Class II or DNA transposons) in the process of transposition, has rendered MITEs as a major cause for natural allelic diversity within or adjacent to plant genes [39,46]. The rice endogenous MITE *mPing *is the most active TE so far documented in any organism, and hence, provides an ideal system for studying the cellular mechanism controlling a TE's activity, as well as a tool for elucidating impact of its activity on adjacent genes. Induced transposition of *mPing *has been firstly discovered independently in three laboratories working with different rice materials, long-term somatic cell cultures of *indica *rice [12], newly initiated anther cultures of *japonica *rice [14], and gamma-ray irradiated *japonica *rice lines [13]. The observation of the sharp difference in the copy numbers of *mPing *between the two cultivated rice subspecies, *indica *and *japonica*, as well as between the two groups of *japonica *cultivars (temperate *vs*. tropical) has led to the suggestion that its transpositional activity has also been induced by other sources of factors. Indeed, it was found that *mPing *can be induced to transpose by interspecific hybridization [18] and hydrostatic pressurization [17]. More recently, it was discovered that in some landraces of *japonica *rice *mPing *has undergone dramatic amplifications associated with domestication and breeding [39], implicating that more potent induction conditions for the element's activity remains to be identified.

Compared with the situation of *japonica *rice, *mPing *activity is less studied in *indica *rice. In this study, somatic cell-derived calli and their regenerants of three rice ssp. *indica *genotypes which are currently under cultivation in large acreages in Burundi and several other African countries showed high frequencies of transpositional activation of *mPing*, though genotypic difference in both excision and insertion frequencies are evident.

Accumulated evidence in various organisms has pointed to the importance of epigenetic modification in the form of cytosine methylation as an important mechanism for repressive control of TEs activity (see Introduction). It is unknown whether alteration in this epigenetic modification has contributed to the activation of *mPing *in any of the hitherto reported cases. Nonetheless, given the inducible nature of *mPing *transposition by various stressful conditions and under which epigentic modifications are known to alter, it is likely that epigenetic mechanisms like cytosine methylation are involved. To address this issue, it is important to have a system wherein both *mPing *activity and alteration in cytosine can be concomitantly induced.

We have shown in this study that various types of cytosine methylation alteration occurred in calli and their regenerants in all three studied rice genotypes, which included both hypo- and hyper-methylation that occurred at CG or CHG sites. Therefore, the tissue culture system (donor seed-plants, calli and regenerants) of these rice ssp. *indica *genotypes provides a system whereby the possible relationship between *mPing *activity and cytosine methylation can be addressed. Indeed, the often-observed phenomenon of somaclonal variation in plant tissue cultures is the results of concerted action of both genetic and epigenetic instabilities induced by the tissue culture process [47], and activity of transposons is known to be involved [47,48]. Furthermore, we recently found that both genetic and epigenetic instabilities in sorghum tissue cultures likely share a common mutagenic basis, as both kinds of instabilities are significantly correlated with disturbed transcript abundance of a set of genes encoding for DNA methyltransferases and 5-methyl-cytosine DNA glycosylases [49]. Therefore, it is reasonable to deduce that there might exist some intrinsic relationships between tissue culture-induced *mPing *activation and perturbed patterns or levels in cytosine methylation in the rice genome.

As a first step to address this issue, we have demonstrated in this study that tissue culture-induced *mPing *activity is indeed correlated with alteration in cytosine methylation at randomly sampled loci across the genome. However, this correlation is cryptic, and which becomes evident only when both the *mPing *activity and methylation alteration are further dissected into more specific aspects. Under such conditions, significant correlations were detected between *mPing *activity and specific types of cytosine methylation alteration both within each genotype and when all three genotypes were considered as a whole.

Recent studies by transposon-methylation display (TMD) showed that in the rice genome various TEs (including *mPing*) appeared to reside in genomic regions with different degrees of cytosine methylation modifications [50]. Thus, given the relationship between the *mPing *activity and alteration in cytosine methylation at globally sampled random genomic loci, discussed above, it is pertinent to ask two questions: (1) Are the genomic regions immediately flanking the *mPing *copies also underwent methylation alteration? (2) Are the methylation states of the *mPing *immediate flanking regions important for the element's stability? To answer these two questions, we performed TMD analysis targeting at the various immobile *mPing *copies in the calli and regenerants from each of the three rice genotypes. We found that both the 5'- and 3'-*mPing *flanks underwent extensive methylation alteration that could also be classified into four types as in the case of random genomic loci detected by MSAP. However, a striking feature characterizing the methylation alteration of the *mPing *flanks is that a specific type of alteration, i.e., CNG hypermethylation, occurred at markedly higher frequencies relative to that of the random genomic loci, in all three genotypes (Figure 2). We consider this as an interesting observation, as it suggests that, specifically at the *mPing*-flanks, loss of CG methylation (commonly occur in tissue culture) was accompanied by rapid hypermethylation of CNG. Given that only the genomic regions flanking the immobile *mPing *copies were analyzed by the TMD method, this observation may implicate that one factor for ensuring stability of the immobile *mPing *copies is due to the rapid CHG hypermethylation which might have been capable of compensating for the almost inevitable loss of CG methylation during callus culture (Figure 2). Indeed, the *mPing *immobility was found as positively correlated only with CNG methylation levels of the 5'-flanking regions but not with that of the 3'-flanks (Table 2). Conceivably, methylation status of the 5'-flanking regions is likely more important for maintaining stability of a TE. Thus, the correlation results are fully supportive for the above speculation that rapid CHG hypermethylation probably have played an important role in maintaining immobility of these *mPing *copies. Indeed, this possibility was further bolstered by the bisulfite genomic sequencing data for an immobile *mPing*-containing locus and an active one. This experiment showed that in callus whereas in the immobile locus loss of CG methylation in the 5'-flank was accompanied by an increase in CHG methylation, such a simultaneous loss of CG and gain of CHG methylation did not occur in the active *mPing*-containing locus; instead, at this locus only loss of CG methylation and no gain of CHG methylation were observed, which though was accompanied with a slight gain of CHH methylation.

The bisulfite sequencing data further suggested that cytosine methylation alteration in the *mPing*-body regions is also likely playing a role in the element's activity or immobility, depending on the loci. The general hypermethylation in all three types of cytosine methylation, CG, CHG and CHH, in the immobile *mPing *copy in callus is striking, as the general trend of alteration in cytosine methylation in tissue culture is genome-wide hypomethylation [47]. Therefore, there must be a mechanism protecting loss of methylation from, and even enhancing methylation at, certain genomic regions wherein the immobile *mPing *copies reside. Conceivably, the small interference (si)-RNA-based RNA-directed DNA methylation (RDM) mechanism is most likely responsible [51-53], and which is in accord with the result that it was CHG and CHH methylation that was increased in the callus (see above). Although it was not possible to analyze the methylation status in the body-region of active *mPing *copies (they were excised), it is probably safe to speculate that it would not have undergo similar enhancement in methylation. Although in theory the siRNA-based RDM mechanism should act *in trans*, it is easy to imagine that the different *mPing *copies being residing at different epigenetic chromatin environments [50] may cause differential accessibility to the siRNAs cues, and hence, produce the difference [51-53]. Further studies which perhaps entail the construction of loss-of-function mutants for each of the genes encoding for the various chromatin structure-maintenance enzymes including DNA methyltransferases, 5-methylcytosine DNA glycosylases and proteins involved in the siRNA-biogenesis pathways, are required to establish if there exists any causal relationships between *mPing *activity and alteration in localized and/or more global cytosine methylation modifications in the rice genome.

## Conclusion

To investigate a possible role of cytosine methylation in the transpositional activity of *mPing*, we analyzed the relationship between TD-based *mPing *transpositions and MSAP- or TMD-based alteration in cytosine methylation in callus and regenerants of three *indica *rice genotypes. We found that, on one hand, *mPing *transpositional activity is correlated with alteration in cytosine methylation patterns at randomly sampled genomic loci (revealed by MSAP), and on the other hand,*mPing *stability is positively correlated with methylation levels of genomic regions immediately flanking the immobile *mPing *copies (revealed by TMD). In addition, high frequency of CNG hypermethylation occurred specifically at the genomic regions flanking immobile *mPing *copies, suggesting that this particular type of methylation modification probably plays an immediate role in fortifying local epigenetic control and ensuring *mPing *stability at these loci, while the failure in this fortification at other *mPing*-flanking regions might be associated with the element's transposition. Bisulfite sequencing of a locus containing an immobile *mPing *copy and one containing an active one further bolstered this possibility.

## Methods

### Callus induction, plant regeneration, and genomic DNA extraction

Embryos from mature seeds of three rice, *Oryza sativa *L., ssp. *indica *cultivars (V14, V27 and R09), widely cultivated in Burundi and some other Africa countries, were used as source of calli. Sterilization and culture/subculture conditions were essentially as reported [54] with the following minor modifications: the macro-nutrients of NMB [N6 [55], micronutrients of MS [56] and vitamins of B5 [57] were used, which were supplemented with 2 mg/L 2,4-D (2,4-dichlorophenoxyacetic acid), 1 mg/L NAA (naphthaleneacetic acid), and 0.5 mg/L KT (kinetin). After six months of subculture, one portion of the calli were transferred onto regeneration medium, which was the maintenance medium with different growth regulators, namely, containing 2 mg/L BAP (benzyl aminopurine) and 0.1 mg/L NAA, while another portion was used for genomic DNA extraction. Shoots of about 10 cm in length were dissected and transferred onto a rooting medium containing 1 mg/L NAA and 0.1 mg/L BAP. Intact plantlets were transferred into autoclaved loamy soil mixed with sand, where they were maintained in a greenhouse conditions at ~25°C with mild illumination. The plantlets survived and developed into healthy plants at high frequencies (> 80%).

Genomic DNA was extracted from fully expanded leaves of five randomly selected regenerated plants for each genotype (designated as V14Reg1–5, V27Reg1–5 and R09Reg1–5, respectively), two pools of calli for each genotype (designated as V14Ca1–2, V27Ca1–1, and R09Ca1–2, respectively), and the donor plants (pools plants germinated from five seeds) for each genotype (V14D, V27D and R09D). A modified CTAB (hexadecyltrimethylammonium bromide) protocol [58] was used, and the DNAs were further purified by two-rounds of phenol extractions.

### Transposon Display (TD) and methylation-sensitive amplified polymorphism (MSAP) assays

The calli and regenerants together with their corresponding donor plants were subjected to transposon-display (TD) analysis [59], using nested *mPing-*specific primers together with a primer designed according to the *Mse*I adapter sequences (see Additional file 1). Digestion/ligation reactions were performed using 300 ng of genomic DNA digested with *Mse*I at conditions specified by the supplier (New England Biolabs Inc.). The MSAP (methylation-sensitive amplified polymorphism) analysis was essentially as reported [41] using various primer combinations (see Additional file 1). Amplification products were separated by 6% polyacrylamide gel electrophoresis. Only clear and highly reproducible bands between two technical replications (starting fro the digestion/ligation step) were scored. Rational for scoring the variable methylation patterns was based on differential presence/absence of a particular band in *Hpa*II and *Msp*I digestions (see Additional file 4). A subset of variable bands representing *mPing *excisions or insertions, and different types of alteration in cytosine methylation patterns were excised from the dried polyacrylamide gels and re-amplified with appropriate primers. *mPing *excisions and insertions were then confirmed by a third *mPing*-specific primer (further internal of the other two) in combination with the *Mse*I primer (see Additional file 1). Isolated TD and MSAP bands were then cloned and sequenced. BlastN was performed against the whole genome draft sequence of the *indica *rice genotype 93–11 to determine chromosomal location of active (excisions and insertions) *mPing *copies, and loci showing alteration in cytosine methylation. BlastX was used for homology analysis of the isolated loci.

### Transposon Methylation Display (TMD) analysis

Transposon methylation display (TMD) is a combination of transposon-display (TD) and methylation-sensitive amplified polymorphism (MSAP) [50]. Thus, the *mPing*-TMD was performed essentially as TD, with modifications only in the restriction enzymes used, namely, *Mse*I in TD being replaced by a pair of isoschizomers, *Hpa*II and *Msp*I. Two sets of consecutive *mPing *– specific primers respectively targeting the 5'- and 3'-*mPing *ends were designed and combined with *Hpa*II/*Msp*I adaptor-primers (see Additional file 1). For amplification and silver-based polyacrylamide gel electrophoresis, the same conditions as in TD and MSAP were used [45]. Because of possible confounding effects of *mPing *transpositions (excisions and insertions), only altering patterns in one of the two enzyme digests (*Hpa*II or *Msp*I) but not in both, which can be unequivocally assigned as due to alteration in cytosine methylation were scored (see Additional file 4).

### Data scoring and analysis

In all three studied markers, TD, MSAP and TMD, changes in the calli and/or regenerated plants relative to the donor plants for a given genotype, which were reproducible between two independent experiments, were scored. For TD, bands disappeared from or appeared in calli and/or regenerated plant(s) relative to the donor plants were scored as *mPing *excisions and insertions, respectively (indeed, all being validated by sequencing). For MSAP, the changing patterns were divided into four major types, CG hypomethylation, CG hypermethylation, CNG hypomethylation and CNG hypermethylation for each genotype (see Additional file 4), as detailed in [45]. For TMD, the scoring rational is similar to MSAP, but only those variable bands that occurred in one of the isoschizomer-digestions, and which can be unequivocally assigned as methylation changes, were scored (see Additional file 4).

Possible correlations between *mPing *activity (excisions and insertions, based on the TD data) and alteration in cytosine methylation at random genomic loci (based on the MSAP data) were tested by using the Pearson correlation analysis. By the same method, Possible correlations between *mPing *stability and two types of methylation levels respectively at the 5' and 3' immobile *mPing*-flanking regions (based on the TMD data) were tested. In both cases, the software SPSS 11.5 for Windows, Bivariate Correlation, Two-tailed, Correlation coefficients, Pearson"  was used, and the statistical significance was determined.

### Bisulfite sequencing

Genomic DNA from a pool of calli (V27Ca2), one regenerant (V27Reg5), and their corresponding seed-plant of cv. V27 was modified using the EZ DNA Methylation-Gold kit (Zymo Research, ) according to the manufacturer's instructions. Briefly, 900 μl ddH_2_O, 50 μl M-dissolving buffer and 300 μl M-dilution buffer were added per tube of CT conversion reagent (Zymo Research) prior to use. Then, 130 μl of bisulfite-containing CT conversion reagent was added to 1 μg of DNA in a volume of 20 μl (150 μl total) and mixed, and the samples were then incubated at 98°C for 10 min, and 64°C for 2.5 h. Modified DNA was purified using a Zymo-Spin IC column (Zymo Research) and stored at -20°C until use. The bisulfite sequencing amplifications primer pairs respectively for a locus containing an immobile *mPing *copy (ITDTG8) and a locus containing an active *mPing *(showing excision in callus) (ITDTA6), both being arbitrarily chosen from the sequenced bands isolated from the TD profiles (Figure 1), were designed using the Kismeth program () and are given in Additional file 6. For each PCR amplification, 2 μl of bisulfite-treated DNA was used as template, and the PCR products were cloned into the pMD18-T vector and sequenced. From 7 to 13 clones for each sample (seed-plant, callus and regenerant), which gave quality sequence reads were included in the analysis. The methylation levels expressed as percentage (%) per site for each of the three types of cytosines, CG, CNG and CHH, were calculated by dividing the number of non-converted (methylated) cytosines by the total number of cytosines of each type within the sequenced regions.

## Authors' contributions

FN and CMX carried out major parts of the experiments, analyzed the data and drafted the manuscript. HYW, LLJ and JSP participated in some of the experiments. BL designed the work, participated in analyzing the data and finalized the manuscript. All authors read and approved the final manuscript.

## Supplementary Material

Additional file 1 **List of adapters and primers used in this study**. These include adapters and primers used in MSAP, TD and TMD analysis. Click here for file

Additional file 2 **Characteristics of tissue culture-induced *mPing *excisions in the three rice *ssp*. indica cultivars, V14, V27 and R09**. A total of 10 mPing excision events which occurred in calli and/or some of the regenerated plants in one or more of the three cultivars were identified by mPing-specific transposons-display (TD) and validated by cloning, sequencing and locus-specific PCR amplification. Click here for file

Additional file 3 **Characteristics of isolated target sites flanking de novo *mPing *insertions in callus and regenerated plants of the three rice *ssp*. indica cultivars, V14, V27 and R09**. A total of 30 de novo mPing insertion events which occurred in calli and/or some of the regenerated plants in one or more of the three cultivars were identified by mPing-specific transposons-display (TD) and validated by cloning, sequencing and locus-specific PCR amplification. Click here for file

Additional file 4 **Category of patterns of cytosine methylation alteration induced by tissue culture in the three rice *ssp*. indica cultivars (V14, V27 and R09) detected by MSAP and TMD**. Types of alteration in methylation patterns occurred at random genomic loci (detected by MSAP) and regions flanking the immobile mPing copies (detected by TMD) summarized. Click here for file

Additional file 5 **Characterization of the isolated variable MSAP fragments from the calli and regenerants of the three rice *ssp*. indica cultivars, V14, V27 and R09**. Chromosomal location, predicted homology and restriction map of the isolated variable MSAP fragments from the calli and/or regenerants of the three rice ssp. indica cultivars, V14, V27 and R09. Click here for file

Additional file 6 **Sequences of primer pairs used for PCR amplification from bisulfite-treated genomic DNA of a seed-plant, a pool of calli and a regenerated plant of cv. V27**. Pairs of bisulfite sequencing primers were designed to amplify a locus containing an inactive mPing copy and a locus containing an active mPing copy, respectively. Click here for file

## References

[B1] McClintock B (1984). The significance of responses of the genome to challenge. Science.

[B2] Slotkin RK, Martienssen R (2007). Transposable elements and the epigenetic regulation of the genome. Nature Reviews Genetics.

[B3] Feschotte C, Jiang N, Wessler SR (2002). Plant transposable elements: Where genetics meets genomics. Nature Reviews Genetics.

[B4] Dooner HK, Weil CF (2007). Give-and-take: interactions between DNA transposons and their host plant genomes. Curr Opin Genet Dev.

[B5] Bennetzen JL (2000). Transposable element contributions to plant gene and genome evolution. Plant Molecular Biology.

[B6] Bureau TE, Wessler SR (1994). Mobile inverted-repeat elements of the Tourist family are associated with the genes of many cereal grasses. Proceedings of the National Academy of Sciences of the United States of America.

[B7] Zhang Q, Arbuckle J, Wessler SR (2000). Recent, extensive, and preferential insertion of members of the miniature inverted-repeat transposable element family Heartbreaker into genic regions of maize. Proceedings of the National Academy of Sciences of the United States of America.

[B8] Xu L, Wang L, Liu T, Qian W, Gao Y, An C (2007). Triton, a novel family of miniature inverted-repeat transposable elements (MITEs) in Trichosanthes kirilowii Maximowicz and its effect on gene regulation. Biochem Biophys Res Commun.

[B9] Kimura S, Oyanagi M, Fukuda T, Ohno Y, Hongo C, Itoh Y, Koda T, Ozeki Y (2008). Role of miniature inverted repeat transposable elements inserted into the promoter region of a carrot phenylalanine ammonia-lyase gene and its gene expression. Plant Biotechnology.

[B10] Feng Q, Zhang Y, Hao P, Wang S, Fu G, Huang Y, Li Y, Zhu J, Liu Y, Hu X (2002). Sequence and analysis of rice chromosome 4. Nature.

[B11] Takagi K, Nagano H, Kishima Y, Sano Y (2003). MITE-transposon display efficiently detects polymorphisms among the Oryza AA-genome species. Breeding Science.

[B12] Jiang N, Bao Z, Zhang X, Hirochika H, Eddy SR, McCouch SR, Wessler SR (2003). An active DNA transposon family in rice. Nature.

[B13] Nakazaki T, Okumoto Y, Horibata A, Yamahira S, Teraishi M, Nishida H, Inoue H, Tanisaka T (2003). Mobilization of a transposon in the rice genome. Nature.

[B14] Kikuchi K, Terauchit K, Wada M, Hirano HY (2003). The plant MITE mPing is mobilized in anther culture. Nature.

[B15] Fujino K, Sekiguchi H, Kiguchi T (2005). Identification of an active transposon in intact rice plants. Mol Genet Genomics.

[B16] Jiang N, Feschotte C, Zhang X, Wessler SR (2004). Using rice to understand the origin and amplification of miniature inverted repeat transposable elements (MITEs). Current Opinion in Plant Biology.

[B17] Lin X, Long L, Shan X, Zhang S, Shen S, Liu B (2006). In planta mobilization of mPing and its putative autonomous element Pong in rice by hydrostatic pressurization. Journal of Experimental Botany.

[B18] Shan X, Liu Z, Dong Z, Wang Y, Chen Y, Lin X, Long L, Han F, Dong Y, Liu B (2005). Mobilization of the active MITE transposons mPing and Pong in rice by introgression from wild rice (Zizania latifolia Griseb.). Mol Biol Evol.

[B19] Yang G, Zhang F, Hancock CN, Wessler SR (2007). Transposition of the rice miniature inverted repeat transposable element mPing in Arabidopsis thaliana. Proceedings of the National Academy of Sciences of the United States of America.

[B20] Gruenbaum Y, Naveh-Many R, Cedar H, Razin A (1981). Sequence specificity of methylation in higher plant DNA. Nature.

[B21] Vanyushin BF (2006). DNA methylation in plants. Current topics in microbiology and immunology.

[B22] Yoder JA, Walsh CP, Bestor TH (1997). Cytosine methylation and the ecology of intragenomic parasites. Trends in Genetics.

[B23] Martienssen RA, Colot V (2001). DNA methylation and epigenetic inheritance in plants and filamentous fungi. Science.

[B24] Chandler VLWV (1986). DNA modification of a maize transposable element correlates with loss of activity. Proc Natl Acad Sci USA.

[B25] Wang L, Heinlein M, Kunze R (1996). Methylation pattern of Activator transposase binding sites in maize endosperm. Plant Cell.

[B26] Cui H, Fedoroff NV (2002). Inducible DNA demethylation mediated by the maize Suppressor-mutator transposon-encoded TnpA protein. Plant Cell.

[B27] Ros F, Kunze R (2001). Regulation of Activator/Dissociation transposition by replication and DNA methylation. Genetics.

[B28] Liu B, Wendel JF (2000). Retrotransposon activation followed by rapid repression in introgressed rice plants. Genome.

[B29] Cheng C, Daigen M, Hirochika H (2006). Epigenetic regulation of the rice retrotransposon Tos17. Mol Genet Genomics.

[B30] Ding Y, Wang X, Su L, Zhai J, Cao S, Zhang D, Liu C, Bi Y, Qian Q, Cheng Z (2007). SDG714, a histone H3K9 methyltransferase, is involved in Tos17 DNA methylation and transposition in rice. Plant Cell.

[B31] Miura A, Yonebayashi S, Watanabe K, Toyama T, Shimada H, Kakutani T (2001). Mobilization of transposons by a mutation abolishing full DNA methylation in Arabidopsis. Nature.

[B32] Kato M, Takashima K, Kakutani T (2004). Epigenetic control of CACTA transposon mobility in Arabidopsis thaliana. Genetics.

[B33] Hirochika H, Okamoto H, Kakutani T (2000). Silencing of retrotransposons in Arabidopsis and reactivation by the ddm1 mutation. Plant Cell.

[B34] Jeddeloh JA, Stokes TL, Richards EJ (1999). Maintenance of genomic methylation requires a SWI2/SNF2-like protein. Nature Genetics.

[B35] Kato M, Miura A, Bender J, Jacobsen SE, Kakutani T (2003). Role of CG and non-CG methylation in immobilization of transposons in Arabidopsis. Current Biology.

[B36] Lippman Z, Gendrel AV, Black M, Vaughn MW, Dedhia N, McCombie WR, Lavine K, Mittal V, May B, Kasschau KB (2004). Role of transposable elements in heterochromatin and epigenetic control. Nature.

[B37] Rangwala SH, Richards EJ (2007). Differential epigenetic regulation within an Arabidopsis retroposon family. Genetics.

[B38] Yu J, Hu S, Wang J, Wong GKS, Li S, Liu B, Deng Y, Dai L, Zhou Y, Zhang X (2002). A draft sequence of the rice genome (Oryza sativa L. ssp. indica). Science.

[B39] Naito K, Cho E, Yang G, Campbell MA, Yano K, Okumoto Y, Tanisaka T, Wessler SR (2006). Dramatic amplification of a rice transposable element during recent domestication. Proceedings of the National Academy of Sciences of the United States of America.

[B40] McClelland M, Nelson M, Raschke E (1994). Effect of site-specific modification on restriction endonucleases and DNA modification methyltransferases. Nucleic Acids Research.

[B41] Reyna-Lopez GE, Simpson J, Ruiz-Herrera J (1997). Differences in DNA methylation patterns are detectable during the dimorphic transition of fungi by amplification of restriction polymorphisms. Mol Gen Genet.

[B42] Xiong LZ, Xu CG, Saghai Maroof MA, Zhang Q (1999). Patterns of cytosine methylation in an elite rice hybrid and its parental lines, detected by a methylation-sensitive amplification polymorphism technique. Mol Gen Genet.

[B43] Ashikawa I (2001). Surveying CpG methylation at 5'-CCGG in the genomes of rice cultivars. Plant Molecular Biology.

[B44] Cervera MT, Ruiz-Garcia L, Martinez-Zapater JM (2002). Analysis of DNA methylation in Arabidopsis thaliana based on methylation-sensitive AFLP markers. Mol Genet Genomics.

[B45] Dong ZY, Wang YM, Zhang ZJ, Shen Y, Lin XY, Ou XF, Han FP, Liu B (2006). Extent and pattern of DNA methylation alteration in rice lines derived from introgressive hybridization of rice and Zizania latifolia Griseb. Theor Appl Genet.

[B46] Huang X, Lu G, Zhao Q, Liu X, Han B (2008). Genome-wide analysis of transposon insertion polymorphisms reveals intraspecific variation in cultivated rice. Plant physiology.

[B47] Kaeppler SM, Kaeppler HF, Rhee Y (2000). Epigenetic aspects of somaclonal variation in plants. Plant Molecular Biology.

[B48] Hirochika H, Sugimito K, Otsuki Y, Tsugawa H, Kanda M (1996). Retrotransposon of rice involved in mutations induced by tissue culture. Proc Natl Acad Sci USA.

[B49] Zhang MS, Xu CM, Yan HY, Zhao N, von Wettstein D, Liu B (2009). Limited tissue culture-induced mutations and linked epigenetic modifications in F1 hybrids of sorghum pure lines are accompanied by increased transcription of DNA methyltransferases and 5-methylcytosine glycosylases. Plant Journal.

[B50] Takata M, Kiyohara A, Takasu A, Kishima Y, Ohtsubo H, Sano Y (2007). Rice transposable elements are characterized by various methylation environments in the genome. BMC Genomics.

[B51] Mathieu O, Reinders J, Caikovski M, Smathajitt C, Paszkowski J (2007). Transgenerational Stability of the Arabidopsis Epigenome Is Coordinated by CG Methylation. Cell.

[B52] Mathieu O, Bender J (2004). RNA-directed DNA methylation. Journal of Cell Science.

[B53] Huettel B, Kanno T, Daxinger L, Aufsatz W, Matzke AJM, Matzke M (2006). Endogenous targets of RNA-directed DNA methylation and Pol IV in Arabidopsis. EMBO Journal.

[B54] Liu ZL, Han FP, Tan M, Shan XH, Dong YZ, Wang XZ, Fedak G, Hao S, Liu B (2004). Activation of a rice endogenous retrotransposon Tos17 in tissue culture is accompanied by cytosine demethylation and causes heritable alteration in methylation pattern of flanking genomic regions. Theor Appl Genet.

[B55] Chu CC, Wang CC, Sun CS (1975). Establishment of an efficient medium for anther culture of rice through comparative experiments on the nitrogen sources. Scientia Sinica.

[B56] Murashige T (1962). A revised medium for rapid growth and bio assays with tobacco tissue cultures. Phys Plant.

[B57] Gamborg OL, Miller RA, Ojima K (1968). Nutrient requirements of suspension cultures of soybean root cells. Experimental Cell Research.

[B58] Kidwell KK, Osborn TC, Beckman JS, Osborn TC (1992). Simple plant DNA isolation procedures. In Plant genomes: methods for genetic and physical mapping.

[B59] Broeck D Van den, Maes T, Sauer M, Zethof J, De Keukeleire P, D'Hauw M, Van Montagu M, Gerats T (1998). Transposon Display identifies individual transposable elements in high copy number lines. Plant Journal.

