# Supplementary material for: Tissue culture-induced transpositional activity of mPing is correlated with cytosine methylation in rice
Source: BMC Plant Biol. 2009 Jul 15;9:91. doi: 10.1186/1471-2229-9-91 (PMC2715021; doi:10.1186/1471-2229-9-91)
Supplement: Additional file 1 — List of adapters and primers used in this study. These include adapters and primers used in MSAP, TD and TMD analysis. [file 1471-2229-9-91-S1.doc]

**Additional file 1** List of adapters and primers used in this study

| Adapters and primers | Sequences (5`-3`) and specifications |
| --- | --- |
| Adapters |  |
| *Eco*RI adapter I | 5`-CTCGTAGACTGCGTACC |
| *Eco*RI adapter II | 5`-AATTGGTACGCAGTC |
| *Hpa*II/*Msp*I adapters I | 5`-GATCATGAGTCCTGCT |
| *Hpa*II/*Msp*I adapter II | 5`-CGAGCAGGACTCATGA |
| *Mse*I adapter I | 5`-GACGATGAGTCCTGAG |
| *Mse*I adapter II | 5`-TACTCAGGACTCAT |
| Preselective primers |  |
| *Eco*RI+A | 5`-GACTGCGTACCAATTCA |
| *Hpa*II/*Msp*I +0 | 5`-ATCATGAGTCCTGCTCGG |
| *Mse*I+0 | 5'-GATGAGTCCTGAGTAA |
| TAILmp1 (5` end) | 5`-GTCACTTTGGGGGGTAAACACTG |
| TAILmp1 (3` end) | 5`-CCGGATTTTGGGTACAAATG |
| Selective primers |  |
| 1. *Hpa*II/*Msp*I -TAC | 5'-ATCATGAGTCCTGCTCGGTAC |
| 2. *Hpa*II/*Msp*I -TTG | 5'-ATCATGAGTCCTGCTCGGTTG |
| 3. *Hpa*II/*Msp*I -TGA | 5'-ATCATGAGTCCTGCTCGGTGA |
| 4. *Hpa*II/*Msp*I -TCG | 5'-ATCATGAGTCCTGCTCGGTCG |
| 5. *Hpa*II/*Msp*I -TCC | 5'-ATCATGAGTCCTGCTCGGTCC |
| 6. *Hpa*II/*Msp*I -TCT | 5`-ATCATGAGTCCTGCTCGGTCT |
| 7. *Hpa*II/*Msp*I -TTC | 5`-ATCATGAGTCCTGCTCGGTTC |
| 8. *Hpa*II/*Msp*I -TTA | 5`-ATCATGAGTCCTGCTCGGTTA |
| 9. *Hpa*II/*Msp*I -TGT | 5`-ATCATGAGTCCTGCTCGGTGT |
| 10. *Hpa*II/*Msp*I -TGC | 5`-ATCATGAGTCCTGCTCGGTGC |
| 11. *Mse*I-0+AT | 5'-GATGAGTCCTGAGTAAAT |
| 12. *Mse*I-0+AC | 5'-GATGAGTCCTGAGTAAAC |
| 13. *Mse*I-0+TA | 5'-GATGAGTCCTGAGTAATA |
| 14. *Mse*I-0+TT | 5'-GATGAGTCCTGAGTAATT |
| 15. *Mse*I-0+TC | 5`-GATGAGTCCTGAGTAATC |
| 16. *Mse*I-0+TG | 5`-GATGAGTCCTGAGTAATG |
| 17. *Mse*I-0+GA | 5`-GATGAGTCCTGAGTAAGA |
| 18. *Mse*I-0+AG | 5`-GATGAGTCCTGAGTAAAG |
| *Eco*RI -ATC | 5'-GACTGCGTACCAATTCATC (combined with 1, 2 and 3 primers) |
| *Eco*RI -ACC | 5'-GACTGCGTACCAATTCACC (combined with 2, 1 and 4 primers) |
| *Eco*RI -ACT | 5'-GACTGCGTACCAATTCACT (combined with 4, 1, 5 and 2 primers) |
| *Eco*RI -ACA | 5'-GACTGCGTACCAATTCACA (combined with 1, 2, 4 and 3 primers) |
| *Eco*RI -AGC | 5'-GACTGCGTACCAATTCAGC (combined with 3, 2, and 1 primers) |
| TAILmp2 (5` end) | 5`-TGTGCATGACACACCAGTG (combined with 11-18 primers; used in TD and TMD) |
| TAILmp2 (3` end) | 5`-TCAAAGTGAAACCCCTCCAC (combined with 1-10 primers; used in TMD) |
| TAILmp3 (5` end) | 5`-CAGTGAACCCCCATTGTGAC (combined with 11-18; 1-10 primers; for further validation of TD and TMD) |
