# Supplementary material for: Tissue culture-induced transpositional activity of mPing is correlated with cytosine methylation in rice
Source: BMC Plant Biol. 2009 Jul 15;9:91. doi: 10.1186/1471-2229-9-91 (PMC2715021; doi:10.1186/1471-2229-9-91)
Supplement: Additional file 2 — Characteristics of tissue culture-induced mPing excisions in the three rice ssp. indica cultivars, V14, V27 and R09. A total of 10 mPing excision events which occurred in calli and/or some of the regenerated plants in one or more of the three cultivars were identified by mPing-specific transposons-display (TD) and validated by cloning, sequencing and locus-specific PCR amplification. [file 1471-2229-9-91-S2.doc]

**Additional file 2**  Characteristics of nine isolated *mPing* excisions in the three rice ssp. *indica* cultivars, V14, V27 and R09

| Locus | Chr. | Locus-specific primer (5’-3’) | Cultivar | Excision Footprint |
| --- | --- | --- | --- | --- |
| ITDTG4 | 3 | For: GCGCAATTCGTACTAGTCGTC  Rev: CATCAAAATCATCGGTGCTG | V14 | agttgccggtttaTAA<mPing (430bp)>TAAtgctctcccttc  agttgccggtttaTAA---------------------------tgctctcccttc |
| ITDTG1 | 2 | For: GCCTTAGTGAACATGGCAGA  Rev: TTGCAGCCAGAGATATCCAA | V27 | gagagtggtgtaTTA<mPing (430bp)>TTAccaaagtatgca  gagagtggtgtaTTA---------------------------ccaaagtatgca |
| ITDGA1 | 2 | For: TGGTAGCTCACGATGGTTAAA  Rev: GCCTTAGTGAACATGGCAGA | V27 | atcggtgcatacttTAA<*mPing* (430bp)>TAAtacaccactctcgg  atcggtgcatactt----------------------------------tacaccactctcgg |
| ITDTG2 | 3 | For: GCTCTCCCTTTTGCTGTTCA  Rev: CATGCCTCCTGCACAGAGTA | V27 | gctctcccttttTAA<mPing (430bp)>TAAtgtgctaaagtg  gctctcccttttTAA----------------------------tgtgctaaagtg |
| ITDTG3 | 3 | For: CTCAAGTTCGCTTGGGAGTC  Rev: TCTCCACCGGAGCTAGGTTA | V27 | acaccagtgTTA<mPing (430bp)>TTAcattaaatg  acaccagtg----------------------------------cattaaatg |
| ITDTG4 | 8 | For: TAGTTCGCACCGTCTCCTTC  Rev: TGCGAGTTGTTTGTGTGGTT | V27 | cactggtgggtcTTA<mPing (430bp)>TTAagcaatatctct  cactggtgggtcTTA----------------------------agcaatatctct |
| ITDTG5 | 5 | For: CGTGTTTTCGTCAACTGGAG  Rev: TGACATGGAAGCAGTAGCAGA | V27 | cactggtgagatTTA<mPing (430bp)>TTAccaccttaagac  cactggtgagatTTA---------------------------ccaccttaagac |
| ITDAC1 | 3 | For: CCCTGCTGCCAAACTTTTT  Rev: TGTGGAATTGTGAGCGGATA | V14 | gtcacaatgggTAA<mPing (430bp)>TAAagagaaaaagt  gtcacaatgggTAA----------------------------agagaaaaagt |
| ITDTA1 | 4 | For: GGGGCTCGCTCTATTTTCTT  Rev: TCCTGTGTGTTGGTCCAGAA | R09 | atttaacatcTAA<mPing (430bp)>TAAaaaatattttct  atttaacatcTAA----------------------------aaaatattttct |
