# Supplementary material for: Tissue culture-induced transpositional activity of mPing is correlated with cytosine methylation in rice
Source: BMC Plant Biol. 2009 Jul 15;9:91. doi: 10.1186/1471-2229-9-91 (PMC2715021; doi:10.1186/1471-2229-9-91)
Supplement: Additional file 3 — Characteristics of isolated target sites flanking de novo mPing insertions in callus and regenerated plants of the three rice ssp. indica cultivars, V14, V27 and R09. A total of 30 de novo mPing insertion events which occurred in calli and/or some of the regenerated plants in one or more of the three cultivars were identified by mPing-specific transposons-display (TD) and validated by cloning, sequencing and locus-specific PCR amplification. [file 1471-2229-9-91-S3.doc]

**Additional file 3** Characteristics of the isolated target sites flanking *de novo mPing* insertions in callus and/or regenerated plants of the three rice ssp. *indica* cultivars, V14, V27 and R09

| Isolated locus | Insertion site ^1^ | | Locus-specific primers (5`-3`) | Isolated from | TIR | TSD |
| --- | --- | --- | --- | --- | --- | --- |
|  | Position | Copy No. |  |  |  |  |
| ITDAC2 | Chr.9 ; AAAA02026645.1; position: 72318 | unique | For: CCATTCCAAACCAGGAGCTA  Rev: TGTGGAATTGTGAGCGGATA | V14Reg2 | GGCCAGTCACAATGG | TTA |
| ITDAC3 | Chr.6;  AAAA02018773.1; position: 28748 | Low | For: TGCGGCTCTAGGAAGTCAAT  Rev: TGTGGAATTGTGAGCGGATA | V14Reg5 | GGCCAGTCACAATGG | TTA |
| ITDAC4 | Chr.7;  AAAA02022617.1; position: 162250 | unique | For: TCAAAAGTGCAAAGCACCAG  Rev: TGTGGAATTGTGAGCGGATA | V27Ca1 | GGCCAGTCACAATGG | TTA |
| ITDAC5 | Chr.11; AAAA02031775.1; position: 42316 | unique | For:  CGGAACGTCGGAAACTAAAG  Rev:  AGTCTACCAAGTGGGGCCTA | V27Ca2 | GGCCAGTCACAATGG | TTA |
| ITDAC6 | Chr.10; AAAA02029526.1; position: 41474 | unique | For: AGGGGATAAACACCCACACA  Rev: GTGAAGGGGGAGATTGTTGA | V27Reg1 | GGCCAGTCACAATGG | TAA |
| ITDAC7 | Chr.3;  AAAA02010758.1; position: 859 | unique | For: AAGTCAGTGGCGACGAAGAT  Rev: ACTGGCCTAAAAGGGAAGGA | R09Reg1 | GGCCAGTCACAATGG | TTA |
| ITDAG2 | Chr.6;  AAAA02020759.1; position: 71248 | Low | For: AGGGGTGATAGGGGAGATTG  Rev: TGAGGAGAAGGGGATTGATG | V27Ca1&  V27Reg4 | GGCCAGTCACAATGG | TTA |
| ITDAG3 | Chr.4;  AAAA02015102.1; position: 20966 | unique | For:  TGGTTAAATAAGACGAATGGTCAA  Rev: CCATTGTGACTGGCCTTAGC | R09Ca1 | GGCCAGTCACAATGG | TAA |
| ITDAG4 | Chr.3;  AAAA02008127.1; position: 34985 | Low | For: TGAGCGAGGCACAAACTATG  Rev: TGTGGAATTGTGAGCGGATA | R09Reg1&  R09Reg2 | GGCCAGTCACAATGG | TAA |
| ITDAG5 | Chr.4;  AAAA02012949.1; position: 17584 | unique | For: TGCGAGAGATGCAAGAGATG  Rev: CTCTCAGACACAAGGCGTGA | R09Reg3 | GGCCAGTCACAATGG | TTA |
| ITDTA1 | Chr.12; AAAA02034216.1; position: 2515 | unique | For: CCTTACGGTTGGCACAATCT  Rev: CCCCAGGCAATCAAGTATTTT | V14Reg1&  V14Reg2 | GGCCAGTCACAATGG | TTA |
| ITDTA4 | Chr.6 ; AAAA02019731.1; position: 42068 | unique | For: TGTGACTGGCCTTAGGGTCT  Rev: GGTAATGATCCACTTGTTCCAGA | V27Reg3 | GGCCAGTCACAATGG | TTA |
| ITDTA5 | Chr.11 ; AAAA02032182.1; position: 34575 | unique | For: CCATTGTGACTGGCCTAATCT  Rev: CATTTTCTTTCGTGAAGCATTG | V27Reg5 | GGCCAGTCACAATGG | TAA |
| ITDAT1 | Chr.5 ; AAAA02016429.1; position: 3713 | unique | For: TGGTTTTTGGGGCACATTAT  Rev: ACGGAGCCACTACGAAGAAA | V14Reg1 | GGCCAGTCACAATGG | TAA |
| ITDAT3 | Chr.3 ; AAAA02008994.1; position: 18039 | unique | For: CCTTTCCTTCCCTCTGGTG  Rev: CGAAAGAAATCAAAAGGAGCA | V14Reg5 | GGCCAGTCACAATGG | TTA |
| ITDAT4 | Chr.1; AAAA02004405.1; position: 13946 | Low | For: GGGGTCGAGATATTCGGTTT  Rev: ACCTTCGGGCAATCCTTTAT | R09Ca1 | GGCCAGTCACAATGG | TTA |
| ITDAT5 | Chr.2;  AAAA02007142.1; position: 15001 | Low | For: GCCAACTTTGATGCCTTCAG  Rev: TGTGGAATTGTGAGCGGATA | R09Ca1 | GGCCAGTCACAATGG | TAA |
| ITDAT6 | Chr.3 ; AAAA02009916.1; position: 107753 | unique | For: TTTACCAAAACTAGTGGAAGTTGTTA  Rev: TGACTGGCCTTAGGTGTTGTC | R09Ca1 | GGCCAGTCACAATGG | TAA |
| ITDAT7 | Chr.9 ; AAAA02027460.1; position: 30613 | unique | For: TCAACGCAGATTCCTTTGTG  Rev: TGTGACTGGCCTAACAGCAG | R09Reg3 | GGCCAGTCACAATGG | TTA |
| ITDTG2 | Chr.12 ; AAAA02033841.1; position: 63428 | unique | For: CTCTCCGTTTGCCTTCTGAT  Rev: AGGCTTGGAGCAGATTACCA | V14Reg2 | GGCCAGTCACAATGG | TTA |
| ITDTG3 | Chr.6 ; AAAA02018380.1; position; 85540 | Low | For: GACTGGCCTTAGGTGTTGTCA  Rev: GGTGAGGAAACACCACATGA | V14Reg3 | GGCCAGTCACAATGG | TTA |
| ITDTG4 | Chr.2 ; AAAA02006856.1; position: 6498 | Low | For: CATTTCAATAGTACGGGTGCTG  Rev: TATGCGTTGCGAACACTTTC | V27Ca1 | GGCCAGTCACAATGG | TAA |
| ITDTG5 | Chr.4;  AAAA02015088.1; position: 29228 | Single | For: CACACACTGCGTCTTGGAGT  Rev: CTGGCCTAATTGGTTTTTGG | V27Ca2 | GGCCAGTCACAATGG | TTA |
| ITDTG6 | Chr.5 ; AAAA02015262.1; position: 26632 | Low | For: GAGTAAATGGGAGGTAGAGGACAA  Rev: AGTTGGTTTGACGCTCGATT | V27Reg1&  V27Reg2 | GGCCAGTCACAATGG | TAA |
| ITDTG7 | Chr.2 ; AAAA02005424.1; position: 24216 | unique | For: GCCTTAGTGAACATGGCAGA  Rev: TTGCAGCCAGAGATATCCAA | V27Reg3 | GGCCAGTCACAATGG | TTA |
| ITDTG9 | Chr.9 ; AAAA02028026.1; position: 44723 | Low | For: ATTCATGTGGGATCGTGACA  Rev: GCTCGTCCACAAGCGAAT | V27Reg5 | GGCCAGTCACAATGG | TAA |
| ITDGA1 | Chr.11 ; AAAA02031083.1; position: 69419 | unique | For: CGCTCCCTTCCCAATACC  Rev: CGTGAGGAATGAGAGCACTG | V14Ca1 | GGCCAGTCACAATGG | TAA |
| ITDGA2 | Chr.12 ; AAAA02033367.1; position: 122910 | Low | For: TTCACCATGACACGCTTGAT  Rev: TGACTGGCCTTACAACCACA | V14Ca1 | GGCCAGTCACAATGG | TAA |
| ITDGA3 | Chr.12 ; AAAA02034927.1; position: 42432 | unique | For: TTTCCAGGAACTAGGACATACCA  Rev: TGACTGGCCTTACAACCACA | V14Reg2 | GGCCAGTCACAATGG | TAA |
| ITDGA4 | Chr.12 ; AAAA02035317.1; position: 35558 | unique | For: TGACTGGCCTTACAACCACA  Rev: TCTAGGAACCAGTGAGAGAAAAA | R09Ca2 | GGCCAGTCACAATGG | TTA |

^1^ Based on BlastN analysis against the whole genome draft sequence of the *indica* rice cv. 9311 at the TIGR Rice Genome Annotation Project - Web BLAST Server
