# Supplementary material for: Tissue culture-induced transpositional activity of mPing is correlated with cytosine methylation in rice
Source: BMC Plant Biol. 2009 Jul 15;9:91. doi: 10.1186/1471-2229-9-91 (PMC2715021; doi:10.1186/1471-2229-9-91)
Supplement: Additional file 4 — Category of patterns of cytosine methylation alteration induced by tissue culture in the three rice ssp. indica cultivars (V14, V27 and R09) detected by MSAP and TMD. Types of alteration in methylation patterns occurred at random genomic loci (detected by MSAP) and regions flanking the immobile mPing copies (detected by TMD) summarized. [file 1471-2229-9-91-S4.doc]

**Additional file 4**  Scored types of alteration in methylation patterns induced by tissue culture at random genomic loci (detected by MSAP) and regions flanking the immobile *mPing*- copies (detected by TMD) in three rice ssp. *indica* cultivars (V14, V27 and R09)

| Banding pattern in donor plant | | Banding pattern in callus and/or regenerated plant | | Type of alteration in cytosine methylation | Scored in MSAP | Scored in TMD |
| --- | --- | --- | --- | --- | --- | --- |
| *Hpa*II | *Msp*I | *Hpa*II | *Msp*I |  |  |  |
| 0 | 1 | 1 | 1 | CG hypomethylation | A1 | C1 |
| 0 | 0 | 1 | 0 | CG hypomethylation | A1 | NC |
| 1 | 0 | 0 | 0 | CG hypermethylation | A2 | NC |
| 1 | 1 | 0 | 1 | CG hypermethylation | A2 | C2 |
| 1 | 0 | 1 | 1 | CHG hypomethylation | B1 | D1 |
| 0 | 0 | 0 | 1 | CHG hypomethylation | B1 | NC |
| 0 | 1 | 0 | 0 | CHG hypermethylation | B2 | NC |
| 1 | 1 | 1 | 0 | CHG hypermethylation | B2 | D2 |

NC: not scored due to possible confounding effects by excision or insertion of *mPing*
