# Supplementary material for: Tissue culture-induced transpositional activity of mPing is correlated with cytosine methylation in rice
Source: BMC Plant Biol. 2009 Jul 15;9:91. doi: 10.1186/1471-2229-9-91 (PMC2715021; doi:10.1186/1471-2229-9-91)
Supplement: Additional file 5 — Characterization of the isolated variable MSAP fragments from the calli and regenerants of the three rice ssp. indica cultivars, V14, V27 and R09. Chromosomal location, predicted homology and restriction map of the isolated variable MSAP fragments from the calli and/or regenerants of the three rice ssp. indica cultivars, V14, V27 and R09. [file 1471-2229-9-91-S5.doc]

**Additional file 5** Chromosomal location, predicted homology and restriction map of the isolated variable MSAP fragments from the calli and/or regenerants of the three rice ssp. *indica* cultivars, V14, V27 and R09

| MSAP segment | Restriction pattern | Chr.  location (BlastN) | Predicted homology  (BlastX) | Size and restriction map |
| --- | --- | --- | --- | --- |
| E2 | E-H fragment | Chr. 1  E-value: e-154 | [gi|34909038|ref|NP_915866.1|](http://www.ncbi.nlm.nih.gov/entrez/query.fcgi?cmd=Retrieve&db=Protein&list_uids=34909038&dopt=GenPept) putative plastid ribosomal protein L34 precursor [*Oryza sativa* (*japonica* cultivar-group)]  3e-23 | EcoRI HpaII  193 121 |
| E6 | E-H fragment | Chr 3  E-value: 6e-76 | LOC_Os06g36830;12006.t03378;unspliced-genomic cysteine synthase, mitochondrial precursor, putative 4e-025 | EcoRI HpaII  112 |
| E7 | E-M fragment | Chr 4  E-value: 3e-045 | LOC_Os04g40540;12004.t03616;unspliced-genomic protein-L-isoaspartate O-methyltransferase, putative, expressed 4e-021 | EcoRI MspI  97 |
| E9 | E-M fragment | Chr. 4  E-value: 1e-055 | LOC_Os08g05750;12008.t00468;unspliced-genomic selenium-binding protein, putative, expressed 2e-024 | EcoRI MspI  21 109 |
| E13 | E-M fragment | Chr. 9  E-value: e-166 | LOC_Os07g04750;12007.t00361;unspliced-genomic F-box domain containing protein 4e-042 | EcoRI MspI  303 |
| E21 | E-H fragment | Chr 9  E-value: 2e-047 | LOC_Os02g22630;12002.t02050;unspliced-genomic conserved hypothetical protein  1e-021 | EcoRI HpaII  113 |
| E22 | E-H fragment | Chr. 9 E-value:  3e-049 | LOC_Os02g22630;12002.t02050;unspliced-genomic conserved hypothetical protein  3e-019 | EcoRI HpaII  117 |
| E24 | E-H fragment | Chr 12  E-value: 1e-064 | [gi|77555856|gb|ABA98652.1|](http://www.ncbi.nlm.nih.gov/entrez/query.fcgi?cmd=Retrieve&db=Protein&list_uids=77555856&dopt=GenPept)  transposon protein, putative, CACTA, En/Spm sub-class [*Oryza sativa* *japonica* cultivar-group]] 2e-08 | EcoRI HpaII  142 |
| E27 | E-M fragment | Chr 12  E-value: 6e-021 | None | EcoRI MspI  62 |
| E29 | E-H fragment | Chr 11  E-value:  e-100 | [gi|77552453|gb|ABA95250.1|](http://www.ncbi.nlm.nih.gov/entrez/query.fcgi?cmd=Retrieve&db=Protein&list_uids=77552453&dopt=GenPept)  hypothetical protein LOC_Os11g44030 [*Oryza sativa* (*japonica* cultivar-group)] 2e-09 | EcoRI HpaII  191 |
| E31 | E-H fragment | Chr 12  E-value: 6.3e-25 | None | EcoRI HpaII  97 |
| E34 | E-H fragment | Chr. 5  E-value: 2e-051 | [gi|53793430|dbj|BAD53153.1|](http://www.ncbi.nlm.nih.gov/entrez/query.fcgi?cmd=Retrieve&db=Protein&list_uids=53793430&dopt=GenPept) hypothetical protein [*Oryza sativa* (*japonica* cultivar-group)] 8e-06 | EcoRI HpaII  41 153 |
| E35 | E-H fragment | Chr 7  E-value:  4e-096 | [gi|50934733|ref|XP_476894.1|](http://www.ncbi.nlm.nih.gov/entrez/query.fcgi?cmd=Retrieve&db=Protein&list_uids=50934733&dopt=GenPept)  unknown protein [*Oryza sativa* (*japonica* cultivar-group)] 6e-15 | EcoRI HpaII  159 36 |
| E36 | E-M fragment | Chr 2  E-value:  3e-081 | None | EcoRI HpaII  110 4 4 56 |
| E39 | E-H fragment | Chr 7  E-value:  3e-039 | None | EcoRI HpaII  94 |
| E40 | E-H fragment | Chr 9  E-value:  5e-006 | None | EcoRI HpaII  67 |
| E46 | E-M fragment | Chr 6  E-value:  3e-093 | LOC_Os06g47834;12006.t04883;unspliced-genomic expressed protein 1e-093 | EcoRI MspI  179 |
| E47 | E-H fragment | Chr 12  E-value: 7.4e-76 | None | EcoRI HpaII  163 |
| E49 | E-H fragment | Chr 5  E-value:  1e-057 | LOC_Os05g05440;12005.t00434;unspliced-genomic expressed protein 5e-058 | EcoRI HpaII  125 |
| E53 | E-H fragment | Chr 9  E-value:  e-109 | None | EcoRI HpaII  257 |
| E54 | E-H fragment | Chr 6  E-value:  4e-096 | [gi|53791942|dbj|BAD54204.1|](http://www.ncbi.nlm.nih.gov/entrez/query.fcgi?cmd=Retrieve&db=Protein&list_uids=53791942&dopt=GenPept)  hypothetical protein [*Oryza sativa* (*japonica* cultivar-group)] 3e-07 | EcoRI HpaII  6 189 |
| E55 | E-M fragment | Chr 1  E-value:  2e-072 | None | EcoRI MspI  96 35 |
| E57 | E-H fragment | Chr. 6  E-value  e-173 | [gi|51090829|dbj|BAD35357.1|](http://www.ncbi.nlm.nih.gov/entrez/query.fcgi?cmd=Retrieve&db=Protein&list_uids=51090829&dopt=GenPept) putative yip1 interacting factor [*Oryza sativa* (*japonica* cultivar-group)] | EcoRI HpaII  334 |
| E58 | E-H fragment | Chr 6  E-value:  e-117 | LOC_Os01g24260;12001.t02148;unspliced-genomic retrotransposon protein, putative, Ty1-copia subclass 3e-011 | EcoRI HpAII  155 71 |
| E73 | E-M fragment | Chr 12  E-value: 1e-064 | LOC_Os12g38040;12012.t03485;unspliced-genomic zinc knuckle family protein, expressed 4e-065 | EcoRI MspI  151 8 |
| E74 | E-H/E-M fragment | Chr 10  E-value: 4e-064 | None | H/M H/M  136 |
| E76 | E-M fragment | Chr 3  E-value:  2e-050 | LOC_Os03g44520;12003.t03839;unspliced-genomic expressed protein 2e-045 | EcoRI MspI  82 37 |
| E77 | E-M fragment | Chr 4  E-value:  1e-020 | LOC_Os04g40540;12004.t03616;unspliced-genomic protein-L-isoaspartate O-methyltransferase, putative, expressed  4e-021 | EcoRI MspI  111 |
| E84 | E-H fragment | Chr 2  E-value:  2e-053 | None | EcoRI HpaII  115 |

Internally methylated CCGG site
