# Supplementary material for: Tissue culture-induced transpositional activity of mPing is correlated with cytosine methylation in rice
Source: BMC Plant Biol. 2009 Jul 15;9:91. doi: 10.1186/1471-2229-9-91 (PMC2715021; doi:10.1186/1471-2229-9-91)
Supplement: Additional file 6 — Sequences of primer pairs used for PCR amplification from bisulfite-treated genomic DNA of a seed-plant, a pool of calli and a regenerated plant of cv. V27. Pairs of bisulfite sequencing primers were designed to amplify a locus containing an inactive mPing copy and a locus containing an active mPing copy, respectively. [file 1471-2229-9-91-S6.doc]

**Additional file 6** Sequences of primer pairs used for PCR amplification from bisulfite-treated

genomic DNA of a seed-plant, a pool of calli and a regenerated plant of cv. V27

| *mPing*-  containing locus | Analyzed plant sample | Primer sequence | Product  size (bp) |
| --- | --- | --- | --- |
| ITDTG8 (immobile) | Seed-plant (V27), callus (V27Ca2) and regenerated plant (V275) | Forward: 5’-yaaagggagtagyyattaagggagagtata-3’  Reverse: 5’-tcaattatttttaraattcarrttaacatara-3’ | 600 |
| ITDTA6  (active) | Seed plant (V27)  (Before excision) | Forward: 5’-gtayatgyaggyyaaattggyaagtagtaa-3’  Reverse: 5’-aatraaatcttttatraaacaatccccaca-3’ | 550 |
|  | Callus (V27Ca2) and regenerant (V27Reg5)  (After excision) | Forward: 5’-gtayatgyaggyyaaattggyaagtagtaa-3’  Reverse: 5’-aataatrrtrtaatcaaarttaacratctct-3’ | 300 |
